# Necrosis avascular bilateral de la cabeza del fémur

**DOI:** 10.7705/biomedica.8133

**Published:** 2026-10-16

**Authors:** Daniel Andrés Nieva-Posso, Leidy Vanessa Imbachi, José Mauricio Ocampo-Chaparro, Daniel Alberto Gutiérrez-Bolaños, Carlos A. Reyes-Ortiz

**Affiliations:** 1 Grupo de Investigación UROGIV, Escuela de Medicina, Universidad del Valle, Cali, Colombia Universidad del Valle Universidad del Valle Cali Colombia; 2 Gestión Clínica y Cuidado Paliativo, Clínica de Alta Complejidad Santa Bárbara, Palmira, Colombia Clínica de Alta Complejidad Santa Bárbara Clínica de Alta Complejidad Santa Bárbara Palmira Colombia; 3 Departamento de Medicina Familiar, Escuela de Medicina, Universidad del Valle, Cali, Colombia Universidad del Valle Universidad del Valle Cali Colombia; 4 Dirección Científica, Clínica de Alta Complejidad Santa Bárbara, Palmira, Colombia Clínica de Alta Complejidad Santa Bárbara Clínica de Alta Complejidad Santa Bárbara Palmira Colombia; 5 Institute of Public Health, Florida A & M University, Tallahassee, FLA, USA Florida A & M University Florida A & M University Tallahassee FLA USA

**Keywords:** osteonecrosis, fémur, insuficiencia renal crónica, diálisis, artroplastia de reemplazo de cadera, Osteonecrosis, femur, renal insufficiency, chronic, dialysis, arthroplasty, replacement, hip

## Abstract

La necrosis avascular del fémur es una enfermedad caracterizada por la interrupción del flujo sanguíneo a la cabeza femoral, lo que genera muerte ósea y colapso articular.

Se presenta el caso de un hombre de 60 años con enfermedad renal crónica, diabetes mellitus e hipertensión arterial sistémica, que recibía hemodiálisis, sin antecedentes de trauma o de uso de esteroides. El paciente desarrolló un dolor progresivo de cadera y limitación funcional bilateral. En las radiografías, se pudieron evidenciar quistes subcondrales y colapso de ambas cabezas femorales, indicativos de avanzada necrosis avascular de fémur. La combinación de enfermedad renal crónica, hipoxia tisular y alteraciones metabólicas, probablemente, contribuyó al desarrollo de la osteonecrosis. Se recomendó artroplastia total de cadera en dos tiempos.

Este caso resalta la importancia de considerar este tipo de necrosis en los pacientes en hemodiálisis y con dolor de cadera no traumático, especialmente, dada la presentación bilateral, poco frecuente y de gran impacto funcional.

## Abstract (en)

Avascular necrosis of the femoral head results from disrupted blood supply leading to bone death and joint collapse.

We report the case of a 60-year-old man with chronic kidney disease on hemodialysis, with diabetes mellitus and hypertension, without trauma or corticosteroid exposure.

He presented with progressive bilateral hip pain and functional limitation. Radiographs revealed subchondral cysts and bilateral femoral head collapse consistent with advanced avascular necrosis of the femoral head. Chronic kidney disease-related hypoxia, metabolic abnormalities, and uremic toxins likely contributed to osteonecrosis. Bilateral total hip arthroplasty in staged procedures was recommended.

This case emphasizes the need to consider this type of necrosis in hemodialysis patients presenting with non-traumatic hip pain, especially given the rare and clinically significant bilateral involvement, which severely impacts mobility and quality of life.

La necrosis avascular del fémur, también conocida como osteonecrosis de la cabeza femoral, es una enfermedad producida por la interrupción del flujo sanguíneo al hueso, lo que provoca la muerte de las células óseas y el colapso progresivo de la articulación coxofemoral ^1^. Se trata de una condición debilitante que puede llevar a una artrosis secundaria y a la necesidad de una artroplastia total de cadera, si no se diagnostica y trata oportunamente.

Las causas de la necrosis avascular del fémur pueden ser multifactoriales, incluyendo traumatismos, uso prolongado de corticosteroides, consumo excesivo de alcohol, enfermedades autoinmunitarias, trastornos de la coagulación, enfermedad renal crónica o causas idiopáticas ^2^. Su diagnóstico se basa en la combinación de la presentación clínica y los estudios imagenológicos; la resonancia magnética es el examen más sensible en las etapas tempranas.

## Presentación del caso

Se presenta el caso de un paciente de sexo masculino de 60 años, con antecedentes de enfermedad renal crónica en estadio 5, que recibió manejo inicial con diálisis peritoneal desde dos años antes y que se encontraba en hemodiálisis desde hacía seis meses. Entre sus comorbilidades, se encuentran la diabetes mellitus con complicaciones microvasculares por neuropatía diabética y enfermedad renal diabética, la hipertensión arterial sistémica, y el síndrome de ansiedad y depresión.

El paciente negó el consumo previo de esteroides, o de caídas o traumas en la región de las caderas. Manifestó presentar un cuadro de seis meses de dolor y limitación funcional progresiva en ambas caderas, que le dificultaban la marcha.

En el examen físico, las maniobras de flexión, aducción y rotación interna y externa -ambas en decúbito supino- generaban dolor en la región inguinal. Se le practicó estudio radiográfico en el que se observó lucidez geográfica irregular y quistes subcondrales en ambas cabezas femorales, con colapso subcondral bilateral de la cortical, con deformidad y aplanamiento de ambas cabezas femorales. Se apreció pérdida del espacio articular coxofemoral bilateral, irregularidad y esclerosis de las superficies articulares; además, disminución de la densidad ósea, sin evidencias de fractura (figura 1).

**Figura 1:**
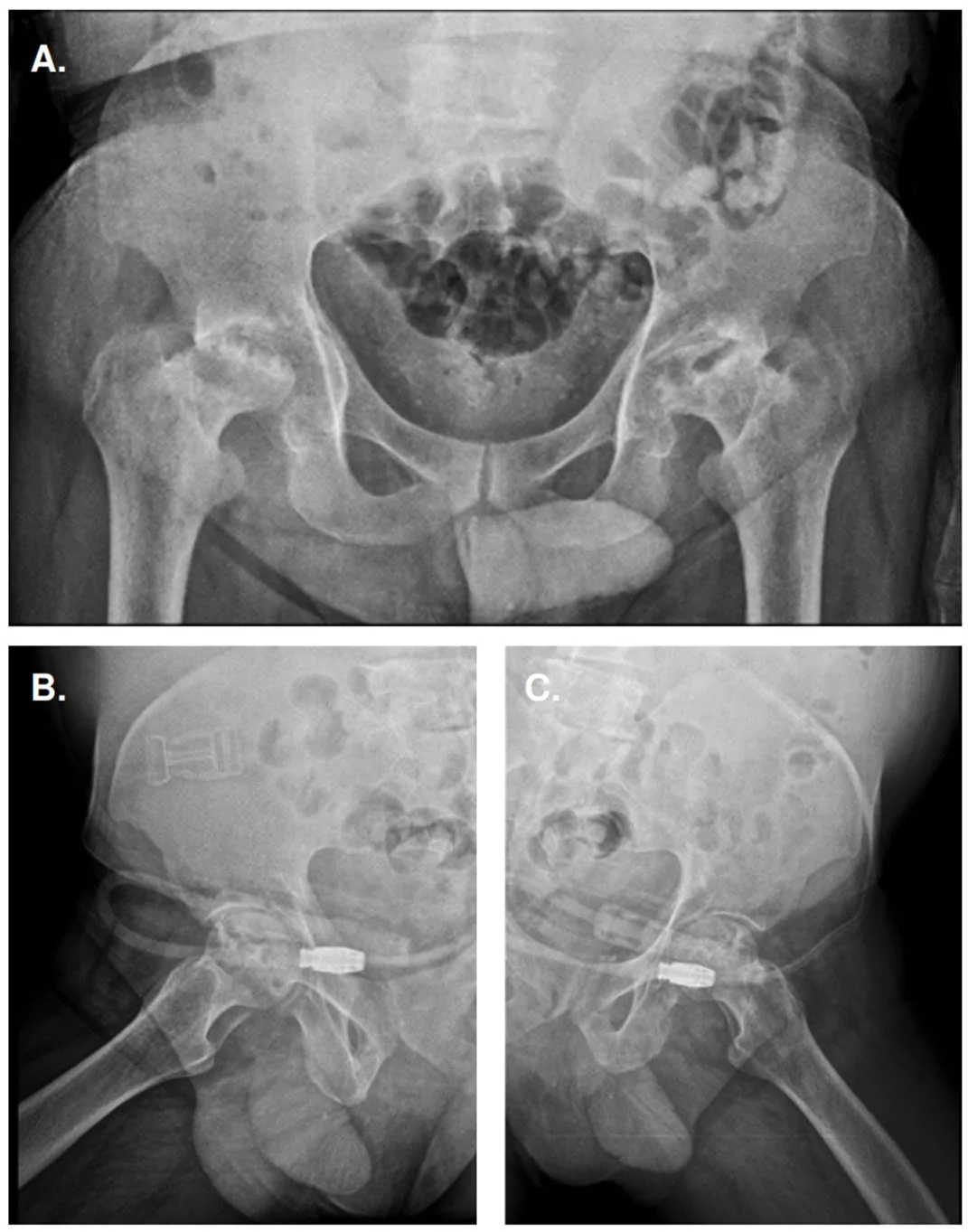
A. Radiografía antero-posterior comparativa de cadera. B. Radiografía oblicua de cadera derecha. C. Radiografía oblicua de cadera izquierda. Se observan la necrosis avascular bilateral de la cabeza femoral, los cambios por coxartrosis y el catéter de diálisis peritoneal.

La necrosis avascular del fémur del paciente se asoció con condiciones predisponentes, como la enfermedad renal crónica y la diabetes mellitus. El paciente se remitió a ortopedia para evaluar un posible reemplazo articular bilateral de cadera, el cual se recomendó que se practicara en dos tiempos quirúrgicos (figura 1).

Este tipo de necrosis en pacientes con enfermedad renal crónica en hemodiálisis es una complicación subestimada, aunque clínicamente significativa. La hipoxia tisular, el hiperparatiroidismo secundario y la acumulación de toxinas urémicas pueden predisponer a la osteonecrosis. En este caso, el paciente presentó un cuadro típico de dolor insidioso y limitación funcional, confirmado por imagenología ^3^.

La necrosis avascular del fémur debe considerarse en los pacientes con enfermedad renal crónica en tratamiento con hemodiálisis, que presentan dolor de cadera sin una causa traumática evidente. La presentación bilateral en las cabezas femorales es inusual y clínicamente relevante, ya que su aparición simultánea compromete de forma significativa la movilidad, la funcionalidad y la calidad de vida del paciente ^4^.

Es fundamental reportar este tipo de casos, no solo por su baja frecuencia, sino también porque permite ampliar el conocimiento sobre sus posibles causas, manifestaciones clínicas y evolución. Además, contribuye a alertar a la comunidad médica sobre la necesidad del diagnóstico temprano y el abordaje terapéutico oportuno, que podrían modificar el pronóstico funcional del paciente y evitar la progresión hacia el colapso femoral bilateral.

### Consideraciones éticas

Aprobado por el Comité de Ética institucional Clínica de Alta Complejidad Santa Bárbara, Palmira, Valle del Cauca, Colombia.
